# Molecular profiling of signaling pathways involved in chicken ovarian follicle development by transcriptome sequencing

**DOI:** 10.3389/fvets.2025.1676247

**Published:** 2026-01-09

**Authors:** Armughan Ahmed Wadood, Farhad Bordbar, Muhammad Safdar, Xiquan Zhang

**Affiliations:** 1 State Key Laboratory of Swine and Poultry Breeding Industry, Guangzhou, Guangdong, China; 2 Guangdong Provincial Key Lab of Agro-Animal Genomics and Molecular Breeding, Key Lab of Chicken Genetics, Breeding and Reproduction, Ministry of Agriculture and Rural Affair, South China Agricultural University, Guangzhou, China; 3 Department of Breeding and Genetics, Cholistan University of Veterinary and Animal Sciences, Bahawalpur, Pakistan

**Keywords:** chicken, follicle, transcriptome, signaling, steroidogenesis, PI3K-Akt, Wnt/*β*-catenin, glycerophospholipid metabolism

## Abstract

Ovarian follicle development in chickens is a dynamic and closely controlled biological process crucial to avian reproduction. Each stage of folliculogenesis is characterized by distinct morphological and molecular alterations, regulated by complex signaling pathways. This study employed transcriptome sequencing to investigate the molecular landscape that governs the transition from primordial (PR) to primary (PM), small white (SW), and small yellow (SY) follicles. We found stage-specific activation of critical signaling pathways involved in follicle growth and development using thorough differential gene expression and pathway enrichment analyses. Our findings showed that the PI3K/AKT/mTOR signaling pathway was highly elevated throughout the shift from PR to PM follicles, highlighting its importance in beginning cellular proliferation and protein synthesis. During development, the Wnt signaling system was regulated from the PM to the SW follicles. This included *β*-catenin-mediated transcriptional regulation, granulosa cell proliferation, and communication between oocytes and somatic cells. Furthermore, the transition from SW to SY follicles was characterized by a significant increase in glycerophospholipid metabolism, emphasizing the metabolic reprogramming required for rapid cellular development and membrane production. The combination of transcriptome data and route mapping provides essential insights into the molecular mechanisms underpinning folliculogenesis in chickens. The signaling pathways revealed the representative stage-specific regulatory networks required for optimal follicle development. These findings enhance our understanding of avian ovarian biology and suggest potential targets for improving reproductive efficiency in chickens.

## Introduction

Reproductive efficiency is crucial for sustaining poultry production (1). In chickens, ovarian follicular development follows a hierarchical progression, allowing for phase-specific study from the dormant PR to the pre-ovulatory stage (2). While follicular dynamics are central to avian reproduction, the molecular mechanisms driving early follicular transitions remain poorly understood (3). Existing studies have focused on hormonal regulation, granulosa cell behavior, and morphology; however, these approaches fall short in elucidating the complex gene regulatory networks involved (4). High-throughput transcriptome sequencing, particularly RNA-Seq, enables a detailed analysis of gene expression and reveals novel transcripts and splicing events critical for follicle maturation (5). However, transcriptomic profiling of early stages, especially PR and PM follicles, is limited in chickens, underscoring the need for targeted studies on early follicular development (6, 7).

In chickens, ovarian follicular development follows a specific sequence, including PR, PM, SW, SY, and pre-ovulatory follicles, each representing a crucial phase in folliculogenesis (8). PR follicles, consisting of a single layer of granulosa cells, constitute the ovarian reserve (9, 10). The initial phase encompasses granulosa cell proliferation, cuboidal cell transformation, and oocyte reactivation (4). SW follicles exhibit heightened transcriptional activity, whereas SY follicles commence yolk buildup and follicle selection (11, 12). Comprehending the molecular signals that facilitate these transitions is crucial for clarifying the mechanisms of recruitment, selection, and differentiation (13). Essential signaling pathways, including the PI3K-Akt, MAPK, Wnt, TGF-*β*, and Notch pathways, govern processes such as cell proliferation, apoptosis, adhesion, and steroidogenesis by integrating hormonal (e.g., FSH, LH) and intrinsic signals (14). Mammalian studies have associated PI3K-Akt with follicle activation and TGF-*β* members, such as GDF9 and BMP15, with granulosa cell function (15); however, their roles in avian folliculogenesis remain inadequately characterized. While the PI3K-Akt and Wnt pathways have been recognized in chicken ovaries (15, 16), the stage-specific and temporal expression data remain insufficient.

Transcriptomic analysis at specific phases of follicular development facilitates the discovery of gene expression dynamics and signaling networks involved in folliculogenesis (16). Comparing the transcriptomes of PR, PM, SW, and follicles enables the mapping of the molecular landscape of follicular advancement and the identification of critical regulatory genes and pathways that facilitate developmental changes (17). This method enhances our understanding of chicken ovarian biology and identifies potential indicators and molecular targets for genetic enhancement or biotechnological intervention to optimize reproductive efficiency (18, 19). Moreover, these analyses provide insights into conserved and species-specific mechanisms relevant to comparative reproductive biology (20).

This study utilized high-throughput RNA sequencing (RNA-Seq) to elucidate transcriptome patterns during the four early follicular phases of chickens. We discovered essential signaling pathways and molecular regulators with stage-specific expression patterns by differential gene expression analysis, functional annotation, and pathway enrichment. Emphasis was placed on cellular development, differentiation, extracellular matrix remodeling, and intercellular communication processes. These findings establish a basis for forthcoming functional investigations aimed at manipulating follicular dynamics to enhance reproductive efficiency in poultry.

## Materials and methods

### Animals and sample collection

Healthy adult laying hens (Chinese Xinghua chicken, *Gallus gallus domesticus*), aged 25 to 30 weeks and kept under standard poultry house conditions, were chosen for this study. Birds were euthanised using cervical dislocation, adhering to institutional animal care and ethical guidelines. Following euthanasia, the ovaries were separated, placed in ice-cold phosphate-buffered saline (PBS), and transported to the laboratory for processing.

### Follicle classification and isolation

**Figure 1:**
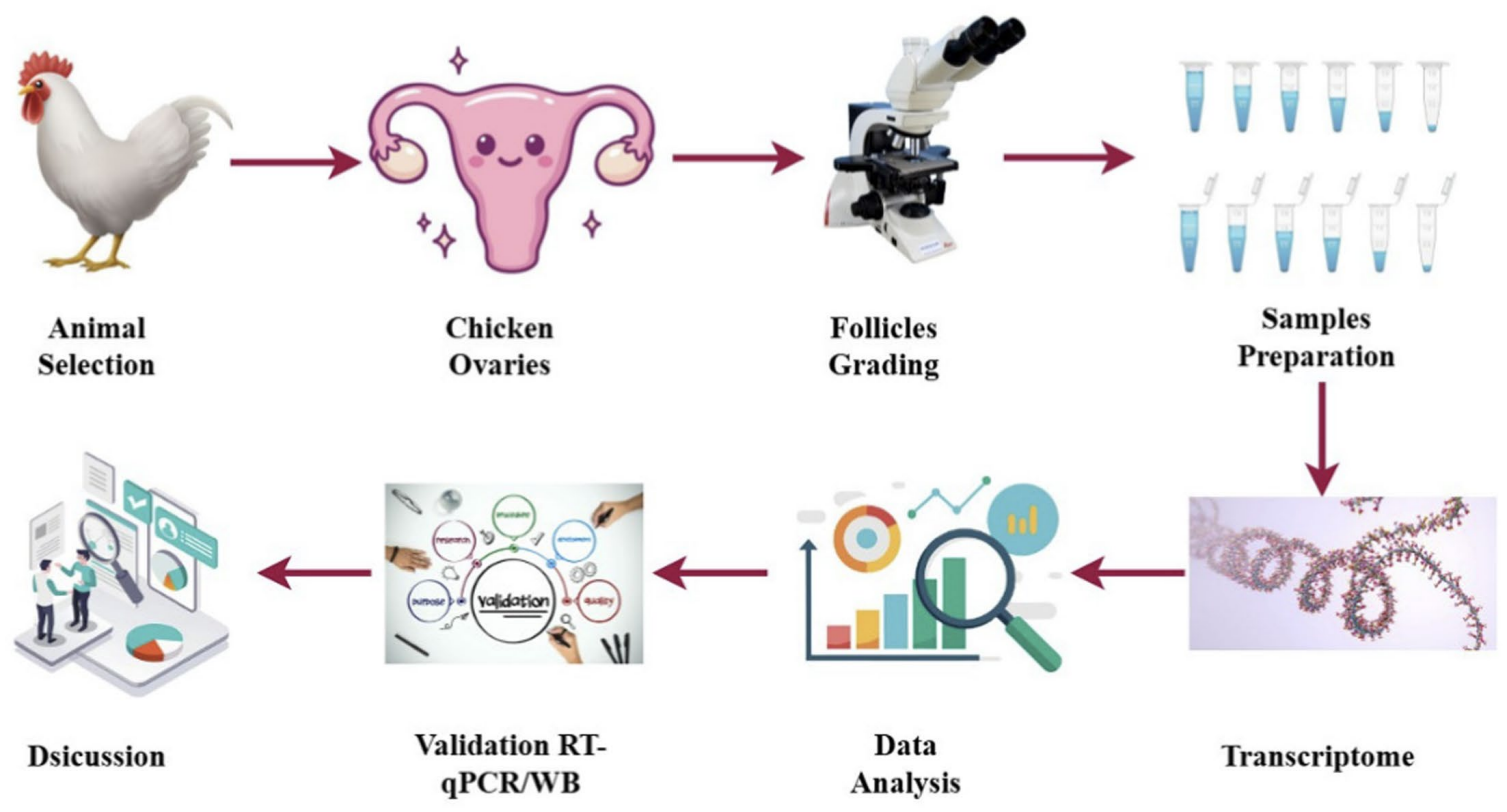
Graphical summary of the experimental workflow used in the study. The process involves animal selection and the collection of chicken ovaries, as well as follicle grading and sample preparation, followed by transcriptome sequencing. Data analysis and validation using RT-qPCR and Western blot (WB) led to the interpretation and discussion of signaling pathways involved in follicle development.

Follicles were dissected using a stereomicroscope and classified into four developmental stages according to size, pigmentation, and morphology: PR follicles (PF, <80 μm, characterized by a single layer of flattened granulosa cells), PM follicles (PR, 80–1,000 μm, featuring cuboidal granulosa cells), SW follicles (SWF, 2–4 mm, translucent), and SY follicles (SYF, 4–6 mm, yellow-pigmented). Follicles from three distinct animals were combined for each category to establish biological triplicates (Figure 1). For the transcriptome analysis, we collected three biological replicates from each category, with each replicate consisting of 12 chicken follicles.

### RNA extraction and quality assessment

Following established protocols, total RNA was extracted from each follicular stage utilizing TRIzol reagent (Invitrogen, USA). Follicle tissues were homogenized in TRIzol, followed by RNA extraction via phase separation using chloroform, isopropanol precipitation, and ethanol washing. The RNA pellets obtained were resuspended in RNase-free water. RNA purity and concentration were assessed with a NanoDrop spectrophotometer, while RNA integrity was verified using an Agilent 2,100 Bioanalyzer. Samples with RIN values exceeding 7.0 were selected for sequencing.

### Transcriptome library preparation and RNA sequencing

RNA samples from each follicular group, with three replicates, were used to construct sequencing libraries employing the NEBNext Ultra RNA Library Prep Kit from New England Biolabs. Messenger RNA was isolated using oligo(dT) magnetic beads and subsequently fragmented, resulting in the synthesis of both the first and second complementary DNA (cDNA) strands. Subsequent steps involved end repair, adaptor ligation, and PCR amplification. The quality and quantity of the library were assessed through Qubit fluorometry and Bioanalyzer analysis. Sequencing was conducted using the Illumina NovaSeq 6,000 platform, yielding 150 bp paired-end reads and an average of 40 million clean reads per sample.

### Transcriptome data processing and DEG analysis

Raw sequencing data were first evaluated with FastQC and subsequently processed with Trimmomatic to eliminate low-quality reads and adaptors. Clean reads were aligned to the *Gallus gallus* reference genome (GRCg6a) utilizing HISAT2. Transcript assembly and expression quantification were conducted using StringTie, with gene expression measured in FPKM units. Differentially expressed genes (DEGs) were identified using DESeq2, with a threshold of |log2 fold change| ≥ 1 and an adjusted *p*-value < 0.05. Gene expression patterns were analyzed using Venn diagrams, hierarchical clustering heatmaps, volcano plots, and principal component analysis (PCA) in R.

### Gene ontology and KEGG pathway enrichment

The ClusterProfiler package in R was used to perform functional enrichment analysis on differentially expressed genes (DEGs). Gene Ontology (GO) terms are categorized into three main classifications: biological processes (BP), molecular functions (MF), and cellular components (CC). Conversely, the Kyoto Encyclopedia of Genes and Genomes (KEGG) analysis revealed enriched pathways during follicular transitions. Significance was assessed using a Benjamini–Hochberg adjusted *p*-value of less than 0.05. The enrichment results were represented as bar plots, differentiating upregulated (red) and downregulated (blue) gene categories throughout developmental stages.

### Protein–protein interaction (PPI) and hub gene identification

Key regulatory genes were identified by inputting differentially expressed genes (DEGs) into the STRING database to construct protein–protein interaction (PPI) networks. Network files were visualized and analyzed using Cytoscape (v3.9), with hub genes identified through the cytoHubba plugin, utilizing degree centrality as the criterion. Three transition-specific networks were developed for PF to PR, PR to SWF, and SWF to SYF. Nodes exhibiting the highest connectivity were identified as hub genes, signifying their pivotal roles in follicular regulation.

### Signaling pathway modeling

Three core signaling pathways were reconstructed based on transcriptomic and enrichment results: the COL6A1-PTEN-PIP3-AKT pathway, which is active during the transition from PF to PR; the Wnt/*β*-catenin-TCF/LEF pathway, which operates during the PR to SWF stage; and PLB1-mediated glycerophospholipid metabolism, which governs the shift from SWF to SYF. The signaling pathways were illustrated using BioRender, https://app.diagrams.net/#, and https://app.diagrams.net/#, drawing on KEGG data and pertinent literature to depict the dynamic regulation of folliculogenesis (assessed on 20-06-2025).

### Statistical and bioinformatics analysis

The statistical analysis of transcriptome data involved differential gene expression (DGE) analysis utilizing edgeR or DESeq2 to identify significant signaling pathways among the four follicle groups: primordial, primary, small white, and small yellow. Pairwise comparisons of consecutive follicle stages were performed to identify differentially expressed genes (DEGs), with expression data normalized using methods including TMM (Trimmed Mean of M-values) or DESeq2’s median of ratios. The significance threshold for identifying DEGs was set at a *p*-value < 0.05, with the false discovery rate (FDR) controlled using the Benjamini-Hochberg correction. Gene Ontology (GO) enrichment and Kyoto Encyclopedia of Genes and Genomes (KEGG) pathway analysis were used to interpret the biological functions and signaling pathways associated with DEGs. Statistical analyses were conducted using R software, utilizing Bioconductor packages for differential gene expression analysis, normalization, and pathway enrichment, thereby ensuring precise interpretation of transcriptome alterations during follicle development.

### qRT-PCR validation of DEGs

Quantitative real-time PCR (qRT-PCR) was performed on selected differentially expressed genes (DEGs) to validate the transcriptomic data, with a focus on key signaling and metabolic pathways. Each group’s micrograms of total RNA were reverse-transcribed utilizing the PrimeScript RT reagent kit (Takara) (21). Quantitative reverse transcription polymerase chain reaction (qRT-PCR) was performed utilizing SYBR Green PCR Master Mix (Applied Biosystems) on a StepOnePlus Real-Time PCR system. Primer sequences were developed using Primer-BLAST and assessed for amplification efficiency. Expression data were normalized to *β*-actin and analyzed using the 2^–ΔΔCt method (22). All reactions were conducted in triplicate, and statistical analysis was performed using ANOVA, followed by Tukey’s *post hoc* test, with significance established at *p* < 0.05.

## Results

### Follicular characteristics across developmental stages

**Figure 2:**
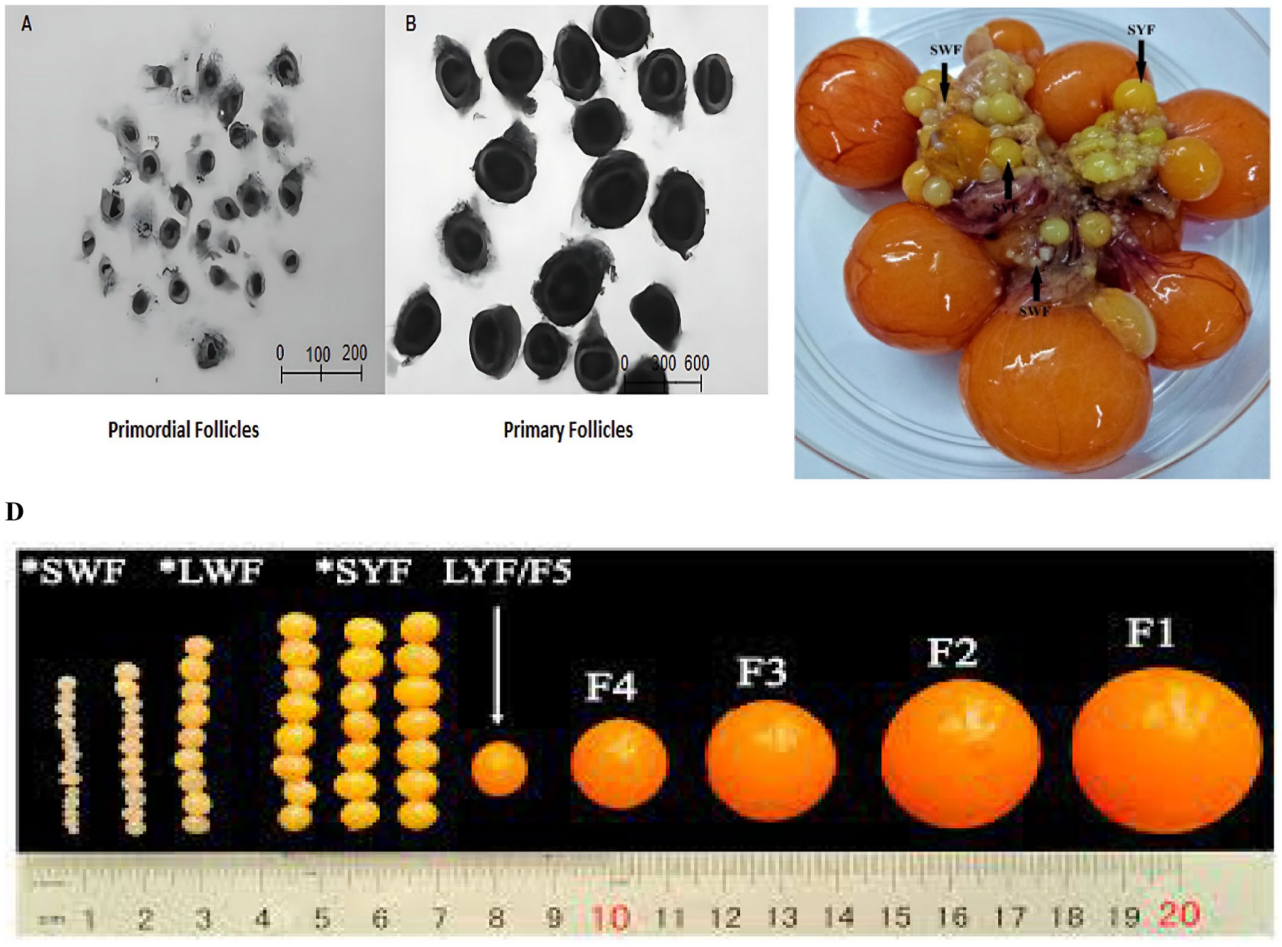
**(A–D)** A morphological comparison between PR and SY follicles. **(A,B)** Representative picture of PR follicles, showing small oocytes surrounded by a single layer of flattened granulosa cells, and panel B represents an image of PM follicles at a more advanced stage, with cuboidal granulosa cells enclosing the oocyte. The images **(A)** were captured with a microscope (50*i*; Nikon, Japan) at 100 × resolution **(C)**. The image depicts SW follicles (SWF) and SY follicles (SYF) in a petri dish. The SYF are bigger and have a characteristic yellow color, indicating advanced follicular maturity. **(D)** The image exhibits a variety of SW follicles (SWF) and SY follicles (SYF), with distinct sizes and structures. The size of the SYF gradually increases as follicular development progresses.

Figures 2A,B display representative microscope images that depict the morphological features of primordial (PR) and primary (PM) follicles extracted from chicken ovaries. Panel A illustrates the PR and PM follicles as small, densely clustered entities situated within a compact ovarian stroma. The follicles generally comprise a centrally positioned oocyte encircled by a single layer of flattened or slightly cuboidal granulosa cells. Their diminutive size, irregular shape, and comparatively low cytoplasmic volume align with the characteristics of early-stage follicular architecture. The observations indicate that the follicles in this group exemplify the earliest and most undifferentiated stages of ovarian development. Panel B illustrates primary follicles, which exhibit a notable increase in size and a more distinct spherical morphology relative to primordial follicles. The oocyte is surrounded by a denser and more uniform layer of granulosa cells, signifying the transition from primordial to early growing follicles. The increased follicular diameter and enhanced cellular boundaries indicate the onset of follicular activation and the commencement of granulosa cell proliferation. The morphological differences between Panels A and B illustrate the structural development occurring in the early stages of chicken folliculogenesis. The images serve as visual confirmation of the staged classification employed for subsequent transcriptomic and functional analyses.

Figure 2C shows SWF and SYF follicles in a petri plate, clearly distinguishing them. SWF are smaller, more transparent, and less mature than SYF, which are larger and yellower, indicating higher lipid buildup. Steroidogenesis increases as follicles yellow, signaling a developmental shift to maturity. The size disparities of these follicles revealed the various stages of follicular development. Figure 2D shows a comparable contrast between SW and SY follicles, showing a visible trend in size. Scaled SYFs are consistently larger and more spherical, indicating a more advanced state of follicular development. The development from early folliculogenesis to a more differentiated state is significant. Additionally, the illustration depicts the growth of SY follicles from F1 to F4, with a visible size gradation across developmental phases. These findings help explain the variation in follicular size and the structural alterations that occur throughout maturation.

### Transcriptome analysis of chicken follicles

**Figure 3:**
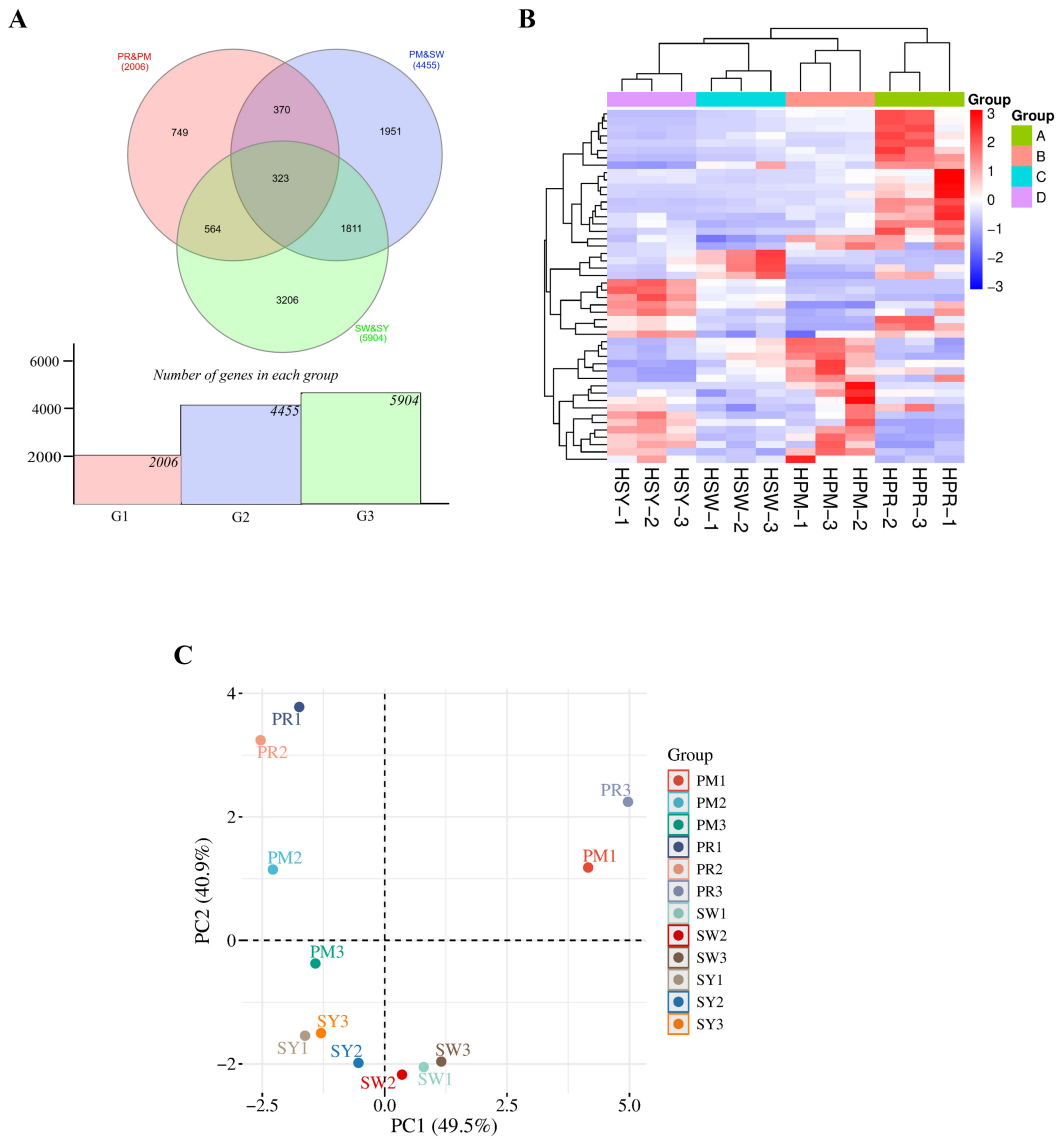
**(A)** Depicts a Venn diagram comparing DEGs across PR vs. PM, PM vs. SW, and SW vs. SY, with 323 genes showing differential expression across all stages. The accompanying bar chart shows how DEG numbers gradually grow as developmental shifts occur. **(B)** Shows a hierarchical clustering heat map that reveals four distinct gene expression patterns and stage-specific changes in gene regulation. **(C)** Describes a PCA plot in which samples are strongly clustered by follicular stage, with PC1 and PC2 accounting for 49.5 and 40.9% of the variance, respectively, showing unique transcriptome profiles and high biological repeatability.

To investigate the molecular dynamics underpinning chicken ovarian follicle development, transcriptome profiling was performed at four critical developmental stages: PR, PM, SW, and SY follicles. The study of differentially expressed genes (DEGs) indicated unique transcriptional alterations between stages. Additional data are presented in Supplementary Tables 1–7, which include genes and corresponding protein accession numbers (DEGs) associated with follicular development. As shown in Figure 3A, the Venn diagram displays 2006 DEGs for PR vs. PM (G1), 4435 for PM vs. SW (G2), and 5904 for SW vs. SY (G3). A core collection of 323 genes showed differential expression across all three transitions, indicating a critical involvement in follicular development. Furthermore, subsets of stage-specific DEGs were discovered, emphasizing the uniqueness of gene expression changes at each transition.

Figure 3B an illustration of hierarchical clustering of the DEGs, which emphasizes stage-specific transcription patterns. The heatmap shows different expression patterns among biological replicates from each follicular stage, indicating reproducible data. Genes were classified into four PM clusters (A-D) based on their expression trends, with significant upregulation or downregulation associated with specific developmental stages. For example, Group A and B genes were primarily expressed in the early stages (PR and PM). In contrast, Group C and D genes were expressed more strongly in the later stages (SW and SY), indicating progressive molecular alterations during follicular growth and selection.

Principal component analysis (PCA) was initially performed to assess the overall quality, intra-group repeatability, and inter-group variation of the follicle samples (Figure 3C). The PCA plot demonstrated distinct separation among the PR, PM, SW, and SY stages along PC1 and PC2, which accounted for 49.5 and 40.9% of the total variance, respectively. Biological replicates exhibited close clustering within each group, demonstrating significant intra-group consistency. To validate the reliability of sample grouping, 95% confidence intervals were estimated for each cluster, showing minimal overlap among the PR, PM, SW, and SY groups. The intervals validated the stability of stage-specific transcriptional patterns and underscored the biological distinctions among the four follicular stages.

Additionally, 95% confidence intervals were computed for each sample cluster on the PCA plot, demonstrating tight clustering within groups and minimal overlap between developmental stages, thus affirming the reliability of the transcriptomic classification.

### Gene ontology pathways enrichment analysis

**Figure 4:**
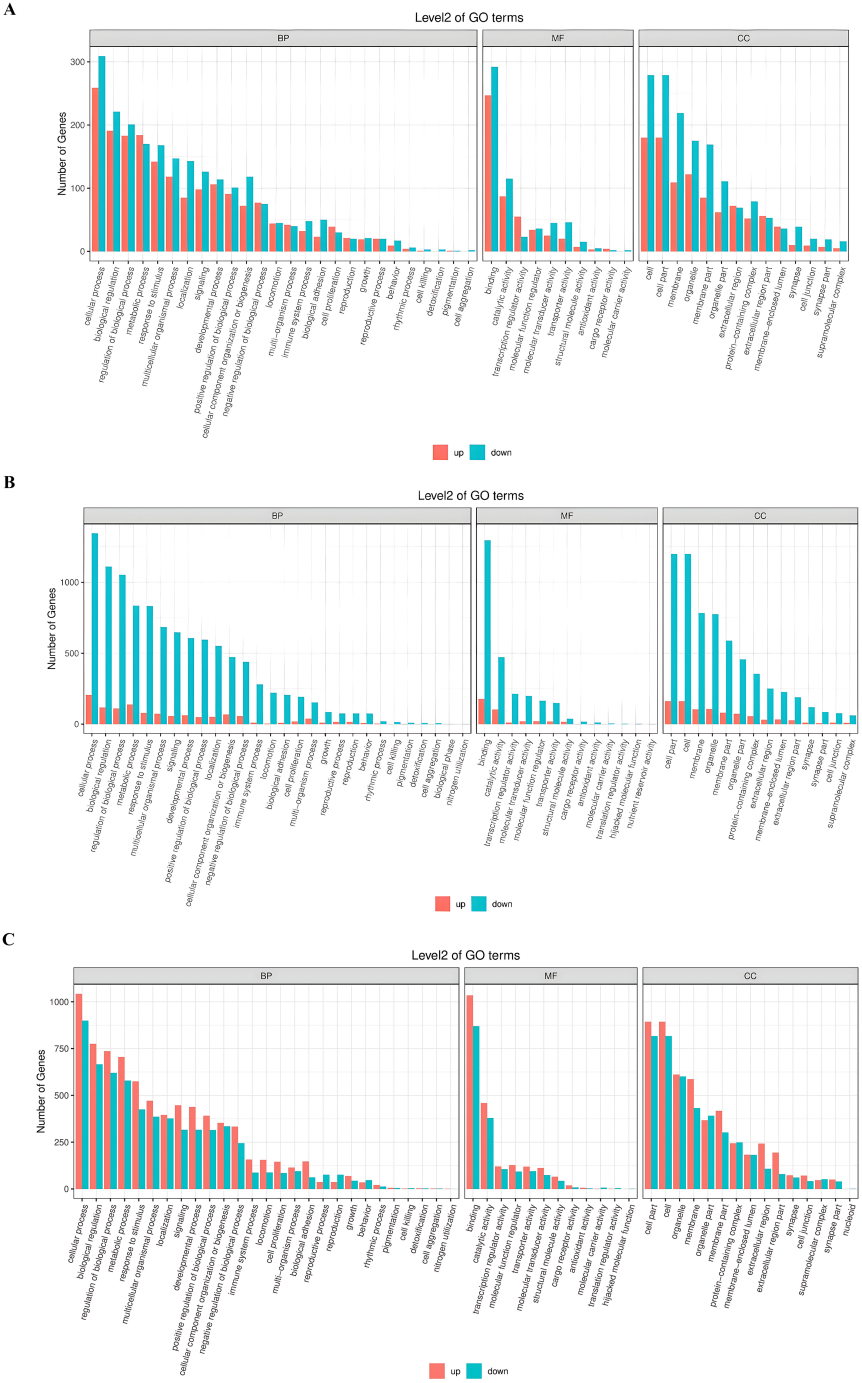
**(A–C)** Gene ontology (GO) examination of the stages of follicular development in chickens. **(A)** Illustrates the shift from PR to PM follicles, **(B)** depicts the development from PM to SW follicles, and **(C)** demonstrates the advancement from SW to SY follicles. The *x*-axis represents the specific GO terms, while the *y*-axis displays the number of genes linked to each phrase. The red bars denote phrases associated with “upregulated” genes, whereas the blue bars indicate those related to “downregulated” genes during the stages of follicular development.

To investigate the functional dynamics underlying chicken ovarian follicle development, we used GO enrichment analysis to identify differentially expressed genes (DEGs) at three key transitions: PR to PM follicles (Figure 4A), PM to SW follicles (Figure 4B), and SW to SY follicles (Figure 4C). The DEGs were divided into three major GO categories: biological process (BP), molecular function (MF), and cellular components. Upregulated genes were primarily associated with cellular responses to stimulation, signal transduction, and developmental processes during the transition from PR to PM follicles. In contrast, downregulated genes were related to metabolic processes and RNA processing. The number of downregulated genes increased significantly from PM to SW follicles stage, particularly in pathways related to translation, ribosome biogenesis, and ribosome structural features, indicating a potential shift in translational control.

In contrast, fewer elevated genes at this stage indicate stronger regulatory control. During the development of SW to SY follicles, both upregulated and downregulated genes helped to achieve a more equal distribution across gene ontology analysis. Upregulated genes were primarily involved in hormone activity, extracellular matrix architecture, and receptor binding, indicating follicular differentiation and hormonal responsiveness. These patterns highlight stage-specific regulatory alterations in gene expression, with early stages characterized by signaling and cellular activation, mid-stages by repression of biosynthetic machinery, and late stages by structural and hormonal specialization.

### KEGG pathways enrichment analysis

**Figure 5:**
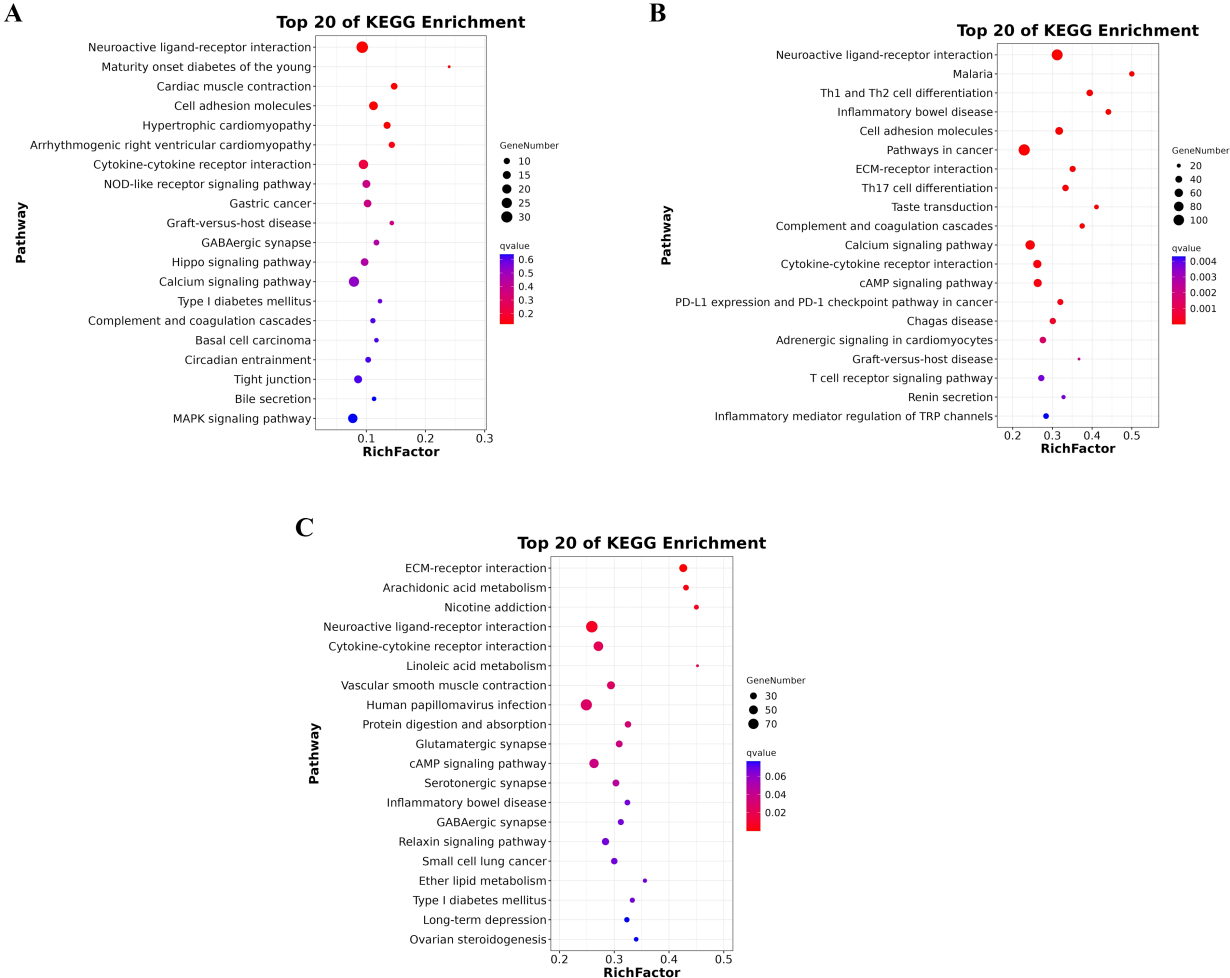
**(A,C)** KEGG pathway enrichment analysis of phases in chicken follicular development. **(A)** Depicts pathways enriched during the shift from PR to PM follicles, **(B)** demonstrates the transition from PM to SW follicles. **(C)** Presents the development from SW to SY follicles. The *x*-axis indicates the number of genes implicated. Simultaneously, pathways are categorized and color-coded according to biological classifications, including metabolism, genetic information processing, cellular activities, organismal systems, and diseases. This study elucidates critical processes governing follicular proliferation across several developmental stages.

KEGG pathway enrichment analysis was employed to examine differentially expressed genes during the transition from PR to SY follicles. Figure 5A illustrates the top 20 KEGG enrichment pathways identified in one of the comparative groups during our transcriptome analysis of chicken ovarian follicle development. The pathway with the highest enrichment was “Neuroactive ligand-receptor interaction,” followed by pathways associated with immune and inflammatory responses, including “Malaria,” “Th1 and Th2 cell differentiation,” and “Inflammatory bowel disease.” Significant pathways related to cellular structure and communication, such as cell adhesion molecules and ECM-receptor interactions, were also highlighted. Additionally, numerous cancer-related pathways, including “Pathways in cancer” and “PD-L1 expression and PD-1 checkpoint pathway,” as well as essential signaling cascades such as “Calcium signaling pathway,” “cAMP signaling pathway,” and “Cytokine-cytokine receptor interaction,” were significantly enriched, indicating their possible involvement in follicular development and regulation.

Figure 5B presents the KEGG enrichment results for an additional comparative group, emphasizing unique metabolic and signaling pathways. The interaction between ECM and receptors was the most enriched, highlighting the significance of extracellular matrix dynamics in follicle maturation. Prominent metabolic pathways such as “Arachidonic acid metabolism,” “Linoleic acid metabolism,” and “Ether lipid metabolism” suggest active lipid-based signaling. Multiple neural and synaptic pathways, including neuroactive ligand-receptor interactions, glutamatergic synapses, and GABAergic synapses, were found to be enriched, in addition to immune-related terms such as inflammatory bowel disease and Type I diabetes mellitus. The presence of the cAMP signaling pathway, Relaxin signaling pathway, and ovarian steroidogenesis indicates their significance in hormonal regulation and steroid production processes essential for ovarian function.

Figure 5C presents the most enriched pathways for a third group, highlighting an emphasis on structural, immune, and disease-specific pathways. Neuroactive ligand-receptor interactions were again prominently featured. The enrichment of cardiac and muscular pathways, specifically “Cardiac muscle contraction” and “Hypertrophic cardiomyopathy,” suggests potential shared regulatory mechanisms with muscle tissue. Significant immune and inflammatory pathways include “Cytokine-cytokine receptor interaction,” “NOD-like receptor signaling pathway,” “Graft-versus-host disease,” and “Type I diabetes mellitus.” Furthermore, pathways regulating cell junctions and polarity, such as cell adhesion molecules and tight junctions, along with significant signaling cascades like the calcium signaling pathway and MAPK signaling pathway, as well as terms related to ovarian steroidogenesis, including bile secretion and circadian entrainment, were identified, providing a thorough overview of the intricate regulatory network involved in folliculogenesis. The three KEGG enrichment analyses collectively illustrate a complex molecular landscape in chicken ovarian follicle development, highlighting neuroendocrine signaling, immune modulation, metabolic regulation, cell adhesion and communication, and canonical developmental pathways, all contributing to follicular growth and maturation.

### Identification of hub genes during follicular development

**Figure 6:**
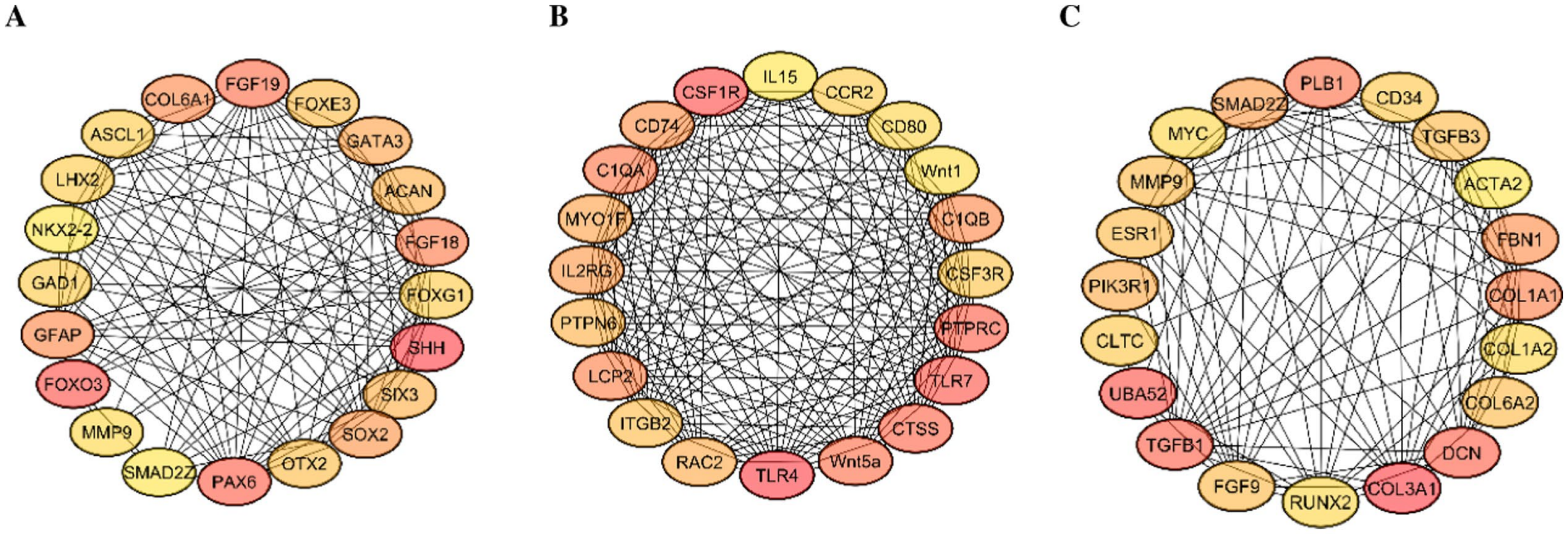
**(A,C)** Identification of hub genes in chicken follicular development phases by Cytoscape software analysis. **(A)** Hub gene network transitioning from PR to PM follicles, **(B)** hub gene network transitioning from PM to SW follicles, and **(C)** from SW to SY follicles. Nodes signify essential genes, with color intensity denoting their level of connectivity (red suggesting greater connectedness and yellow indicating lesser connectivity). Edges represent interactions among genes. These hub genes likely provide essential regulatory functions in follicle activation, growth, and differentiation throughout each of the embryonic phases.

A hub gene network analysis was performed to examine the molecular framework of chicken folliculogenesis across three developmental transitions. The transition from PR to PM follicles (Figure 6A) identified several key transcription factors and signaling molecules with significant connectivity, including COL6A1, FOXE3, and SOX2. The genes are primarily associated with neurogenesis, cell fate determination, and inflammatory signaling, suggesting a significant reprogramming phase during the initial recruitment of follicles. The transition from PM to SW follicles (Figure 6B) involved a shift in gene expression of the Wnt family (Wnt1, Wnt5, Wnt11). These are primarily linked to the modulation of immune responses and Wnt signaling, indicating that immune regulation and morphogenic cues are crucial at this stage. During the transition from SW to yellow follicles (Figure 6C), key genes such as COL1A1, TGFB1, MYC, and PLB1 were predominant in the interaction network. These genes regulate extracellular matrix remodeling, cell proliferation, and hormone responsiveness, which are essential for follicular growth and differentiation. The progressive rewiring of hub gene networks across developmental stages highlights specific regulatory modules that coordinate ovarian follicle development, ranging from initial activation signals to structural and functional specialization.

### Quantitative real-time PCR (qRT-PCR)

**Figure 7:**
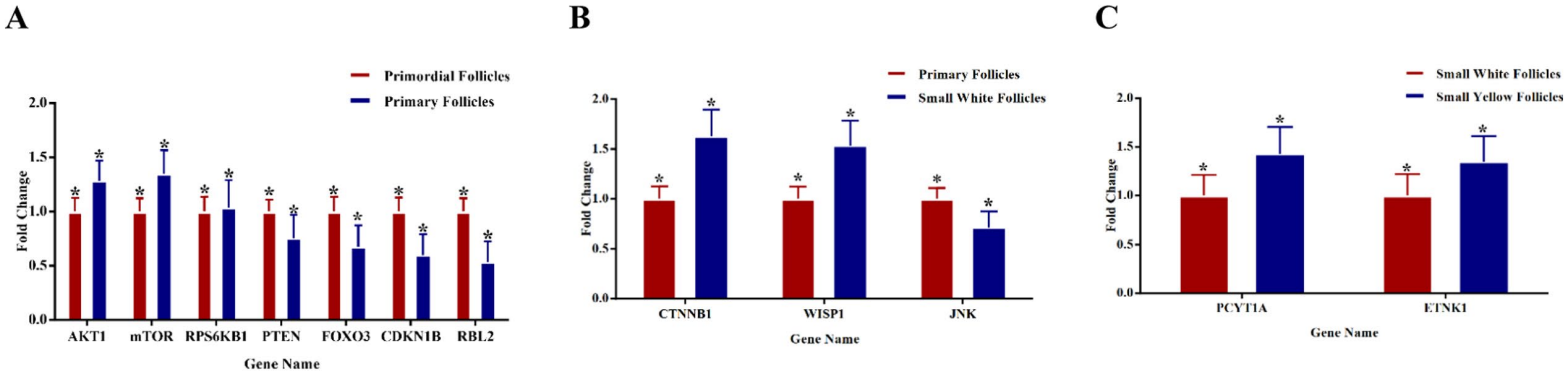
**(A–C)** Quantitative mRNA expression levels of key genes associated with follicular development at various stages of chicken ovarian follicles, as assessed by qRT-PCR. **(A)** Comparison of PR and PM follicles reveals a notable overexpression of AKT1, mTOR, and RPS6KB1, accompanied by a downregulation of PTEN, FOXO3, RBL2, and CDKN1B. **(B)** CTNNB1, WNT1, and JNK expression levels are markedly elevated in SW relative to SY follicles. **(C)** The expression levels of PCYT1A and ETNK1 are increased significantly in SY follicles compared to SW follicles. Data are expressed as mean ± SEM (*n* = 3). Asterisks (*) denote statistically significant differences (*p* < 0.05) among the groups being compared.

We utilized quantitative real-time PCR (qRT-PCR) to analyze the expression of key genes involved in follicular development in chickens at various stages of follicular development (Figures 7A–C). PM follicles increased AKT1, mTOR, and RPS6KB1 levels, while indicating reduced levels of PTEN compared to PR follicles. Activating the PI3K/AKT/mTOR pathway is essential for follicular development. Elevated levels of AKT1 and mTOR and reduced PTEN indicate that this signaling pathway facilitates early follicular growth. CTNNB1, WNT1, and JNK levels were elevated from PM to SW follicles. The elevated expression of CTNNB1 and WNT1 indicates an enhanced Wnt/*β*-catenin signaling pathway, which may promote granulosa cell proliferation and follicular maturation. During this intermediate developmental phase, elevated JNK expression, a gene associated with stress signaling and cellular proliferation, may enhance cellular remodeling or survival signaling. SW and SY follicles demonstrated higher levels of PCYT1A and ETNK1, genes related to the Kennedy pathway for phospholipid synthesis. These gene expression profiles illustrate the essential signaling and metabolic activities involved in follicle development. Stage-specific expression patterns indicate the coordinated control of pathways, such as the PI3K/AKT/mTOR and Wnt/β-catenin pathways, alongside metabolic alterations that facilitate follicular growth and differentiation.

## Discussion

The developmental pathway of chicken ovaries exhibits a distinct evolution in physical characteristics, closely corresponding to the underlying molecular mechanisms (23). PR follicles were revealed to contain small oocytes surrounded by a single layer of granulosa cells, indicating their dormant state (24). Conversely, PM follicles exhibited cuboidal granulosa cells, indicating the initiation of follicular activation (25). The morphological metamorphosis persisted in SW follicles (SWFs) and SY follicles (SYFs), where alterations in follicle size, shape, and color were apparent (23, 26). The structural changes, such as lipid accumulation in SYFs, suggest the initiation of steroidogenesis and prepare the follicle for ovulatory function (27).

The transcriptomic analysis of chicken ovarian follicle development has demonstrated a tightly regulated, stage-specific molecular pathway necessary for folliculogenesis (28, 29). The transition from PR to PM follicles is characterized by the upregulation of differentially expressed genes primarily associated with cell division, proliferation, and signal transduction, indicating the onset of follicular activation (30). The downregulation of lipid metabolism-related terms suggests that energy metabolism is involved in cellular growth and differentiation (31). Increasing the extracellular matrix (ECM), associated with cellular components, facilitates the structural remodeling required for follicle growth (32). KEGG and Gene Ontology enrichment analyses confirmed these findings, highlighting the early-stage activation of key biological processes, including carbohydrate and lipid metabolism, ribosomal function, and the PI3K-Akt and Wnt signaling pathways (33).

Hub gene analysis of follicle development reveals regulatory changes that regulate the folliculogenesis signaling pathways. From PR to PM, genes such as COL6A1, FOXL2, and SHH become important hubs, initiating the COL6A1-PTEN-PIP3-AKT signaling pathway (34). This pathway facilitates cellular growth and anti-apoptotic signals that may support early follicle survival and granulosa cell proliferation (35, 36). During the PM to SW development, hub genes, including Wnt5 (37), LCP2 (38), and TCF7, indicate that the Wnt/*β*-catenin-TCF/LEF signaling axis dominates follicle selection and oocyte-granulosa cell communication. Finally, PLB1 and other metabolism-related genes, such as ALB and GPD1, show metabolic reprogramming, notably via the PLB1-regulated glycerophospholipid metabolism pathway, in SW to SY follicles. This shift promotes energy-intensive membrane biosynthesis (39) and follicular growth. These stage-specific hub gene networks illustrate how signaling and metabolic pathways are coordinated to promote the development of chicken ovarian follicles.

As follicles progressed from PM to SW and subsequently to SY stages, their transcriptomic profiles exhibited increasing specialization. The GO analysis conducted during the PM-to-SW transition highlighted the roles of protein binding (35), hormone regulation (36), and cellular communication (35), aligning with the initiation of meiotic activity and the development of oocyte competence (40). The subsequent development of these follicles indicated an essential shift in steroidogenesis, lipid metabolism, and endocrine regulation (38), highlighting the preparation for ovulation (41). KEGG pathway analysis revealed significant activation of steroid hormone biosynthesis and ECM-receptor interactions (42). Hub gene analysis indicated a biological transition from transcriptional regulation (FOXE3, SOX2) to immune modulation (CD74, TLR7), followed by ECM remodeling (COL1A1, FN1) and growth factor signaling (TGFβ1, FGF9). These findings suggest that follicular development in chickens is governed by a complex interplay of metabolic, hormonal, and structural signals, offering crucial insights into the molecular basis of avian reproductive biology.

### COL6A1/FOXO3-PTEN-PIP3-AKT signaling pathway

**Figure 8:**
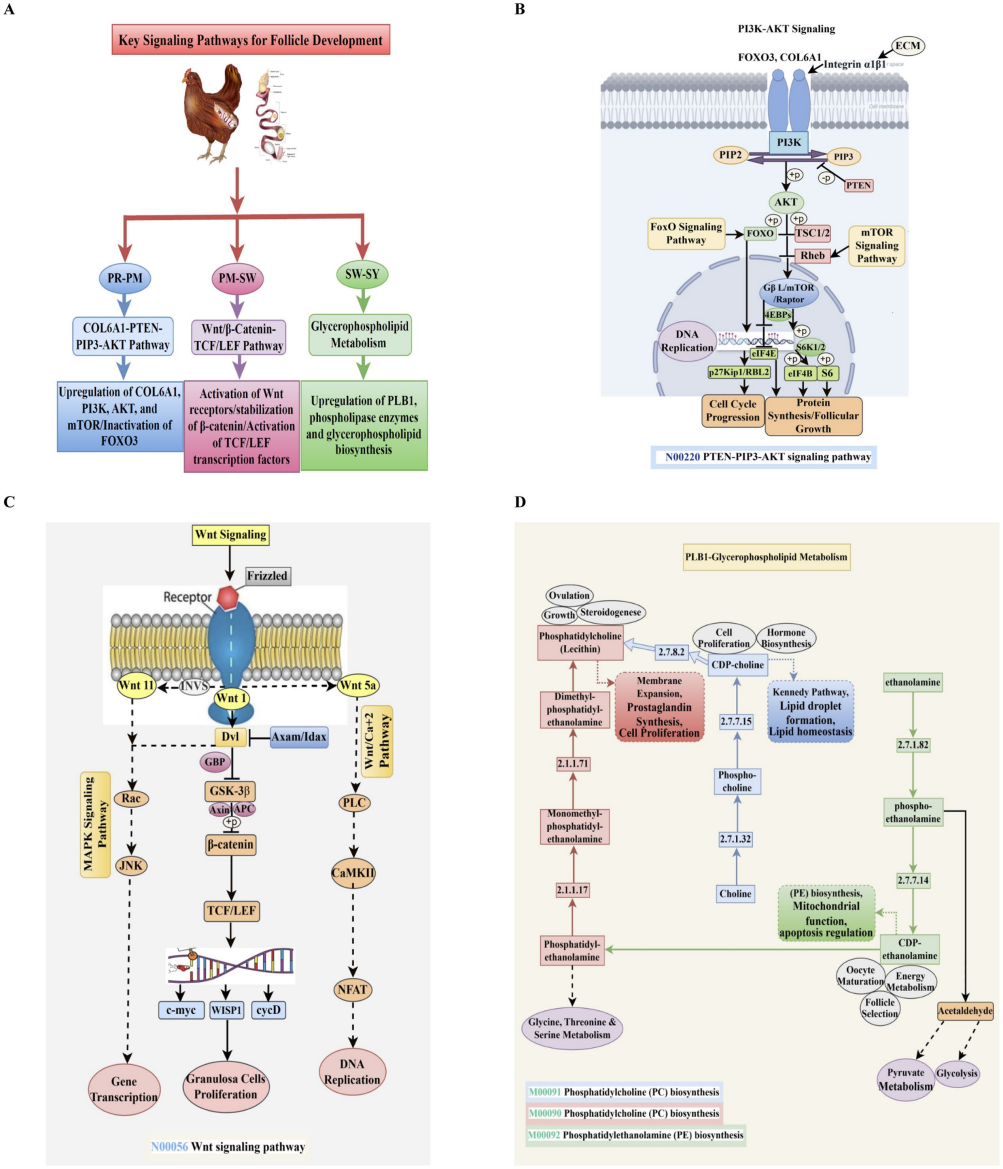
**(A)** Schematic summary figure illustrating the key pathways and their stage-specific activation. **(B)** The COL6A1-PTEN-PIP_3_-AKT pathway signals chicken PR follicles to PM. COL6A1 and FOXO3 engage with the ECM via Integrin α1β1, starting the PI3K/AKT cascade. PI3K transforms PIP_2_ to PIP_3_, activating AKT. PTEN inhibits mTOR signaling by dephosphorylating PIP_3_. **(C)** Wnt/β-catenin-TCF/LEF signaling pathway in PM to SW follicle development. Dvl signal transduction begins when Wnt 1, 3, and 5 bind to Frizzled and LRP5/6 co-receptors. Nuclear translocation and stability are achieved by inhibiting the β-catenin degradation complex (Axin, APC, GSK3β). TCF/LEF transcription factors and β-catenin affect granulosa cell proliferation genes (c-myc, cyclin D1, BIRC5). MAPK, PKC, and PI3K/Akt pathways increase gene expression, proliferation, and DNA replication. **(D)** PLB1-mediated glycerophospholipid metabolism. For lysophospholipids and free fatty acids, phospholipases break down PC, PE, and PI. Both enlarged follicles and steroidogenesis require membrane remodeling, lipid signaling, and energy consumption.

Transitioning from PR to PM follicles is a key step in regulating chicken ovarian folliculogenesis (43, 44). The transition of these follicles involves the PTEN-PI3K-AKT pathway as a key regulatory mechanism (Figures 8A–D). This pathway regulates the activation of PR follicles through downstream effectors that govern protein synthesis and cell survival (45, 46). The current study revealed that genes encoding critical components, including PI3K, AKT, and mTOR, showed upregulation, indicating enhanced mTORC1 activity (Figure 8B). This activation enhances protein synthesis by phosphorylating 4EBP1 and S6K1, subsequently initiating granulosa cell proliferation and oocyte growth (47). The PI3K-AKT pathway regulates the participation the signaling pathways that control follicular development (48). COL6A1, which encodes the α1 chain of type VI collagen, is a structural part of the extracellular matrix (ECM) (49) and acts as a molecular signal for cells (50, 51). COL6A1 interacts with integrin receptors, specifically integrin α1β1, on the surfaces of oocytes and granulosa cells as the follicles grow (52). This connection is crucial for sending signals from the ECM that activate pathways inside cells (53). When COL6A1 binds to integrins, it activates phosphatidylinositol 3-kinase (PI3K) at the cell membrane (54). This enzyme then phosphorylates PIP2 to form PIP3, the first step in a series of signaling events (55).

The transcription factor FOXO3 is inactivated once activated by AKT, which promotes the production of cell cycle inhibitors, including p27Kip1 and RBL2 (56, 57). FOXO3 facilitates the preservation of follicles in their inactive condition. This inhibition is removed upon the inactivation of FOXO3, promoting the proliferation of granulosa cells and the progression of follicular expansion (58, 59). Previous studies have established FOXO3 as a regulator of follicular activation, while PTEN is crucial for modulating AKT activation by inhibiting the conversion of phosphatidylcholine 3,4-bisphosphate (PIP3) to phosphatidylcholine 3,4-bisphosphate (PIP2). This indicates that diminished PTEN expression or activity may lead to premature follicle activation, potentially affecting ovarian reserve maintenance and fertility. PIP3 is a docking site for AKT (protein kinase B), facilitating its phosphorylation and subsequent activation. Activated AKT targets various substrates, influencing cell survival, metabolism, and growth (60). The activation of AKT notably inhibits the TSC1/2 complex, subsequently relieving the suppression of Rheb, a GTPase that activates mTORC1 (mTOR-Raptor complex) (61). The activation of the mTOR pathway enhances protein synthesis and cellular growth by phosphorylating 4EBPs and S6K1/2, which in turn stimulate translation initiation factors, including eIF4E, eIF4B, and ribosomal protein S6 (62). This pathway leads to increased proliferation of follicular cells and growth of oocytes, thereby facilitating the transition to PM follicle growth.

The tumor suppressor PTEN (Phosphatase and TENsin homolog) is a key regulatory checkpoint on this axis (63). It stops PI3K signaling by dephosphorylating PIP3 back to PIP2 (64). This negative regulation ensures that the PI3K-AKT pathway is only activated when needed, maintaining the delicate balance between follicular dormancy and activation. The downregulation of PTEN, a negative regulator of PI3K signaling, provides additional support for the hypothesis of pathway activation during follicle development (65). The temporal changes in PTEN expression in our dataset suggest that it could function as a molecular brake to prevent premature follicle activation, consistent with its known function in mammals (46). The mechanical signals required for follicle activation may be facilitated by type VI collagen and FOXO3 in conjunction with the integrin α1β1 at this stage, suggesting ECM remodeling and integrin signaling (66). When integrins are engaged, PI3K is activated, linking changes in the extracellular matrix to pathways within cells that regulate growth (67). These interactions are essential for the dissolution of the dormancy barrier in PR follicles (68, 69).

Figures 4A, 7A, which combine transcriptome data with KEGG and pathway visualizations, indicate that the COL6A1/FOXO3-PTEN-PIP3-AKT axis functions as part of a more comprehensive network. The PI3K-AKT–mTOR pathway engages multiple signaling networks, including FoxO, NF-κB, and extracellular matrix signaling pathways (70), which together govern essential cellular processes such as apoptosis inhibition, oxidative stress response, and metabolic homeostasis (71, 72). The results highlight the critical importance of the PI3K-AKT–mTOR pathway and extracellular matrix signaling, as mediated by COL6A1 and FOXO3, in the initial phases of chicken follicular development. Transcriptome analysis and pathway enrichment profiling are critical in regulating the ovulation process in chickens, including follicle activation and initial follicular development.

### Wnt/*β*-catenin/TCF-LEF signaling pathway

The development of PM to SW follicles is an essential step in the growth and differentiation of granulosa cells in the chicken ovary. The Wnt/β-catenin/TCF-LEF pathway is activated during this change (73) (Figure 8C). It regulates cell fate, growth, and gene expression (74). This pathway is activated in the present transcriptome profile, indicating its critical role in follicular development and early folliculogenesis. Granulosa cell fate decisions may be influenced by canonical and non-canonical Wnt signaling, as indicated by the upregulation of Wnt receptor expression, which includes Wnt4, Wnt5a, and Wnt11 (75–77). Frizzled receptors and the stability of *β*-catenin drive canonical Wnt signaling, promoting the transcription of target genes linked to proliferation, including cyclin D1, WISP1, and c-myc (78, 79). As a result, the granulosa cells required for follicular wall growth can grow and differentiate more easily (73, 80). Wnt signaling initiates when specific Wnt ligands, including Wnt1, Wnt5, and Wnt11, bind to the Frizzled (Fzd) receptor and its co-receptors located on the cell surface (81). This interaction triggers the activation of the Dishevelled (Dvl) protein, which serves as a crucial hub in the Wnt signal transduction (82). Dvl prevents the phosphorylation and subsequent degradation of *β*-catenin by blocking the degradation complex of Axin, APC, and GSK-3β (83). The result is that β-catenin accumulates in the cytoplasm and then enters the nucleus (84).

Once the nucleus, β-catenin interacts with TCF/LEF transcription factors, activating a range of target genes that play crucial roles in cell proliferation and differentiation (85). This set of genes includes c-myc, WISP1, and cyclin D (cyclin D) genes, which are necessary for granulosa cell proliferation —a feature of functional maturation and follicle expansion during the transition from PM to SW follicle stage (86). These genes work together to facilitate DNA replication, initiate metabolism, and enable the structural reconfiguration necessary for continuous follicular development (87). In addition, the canonical *β*-catenin pathway is influenced by noncanonical Wnt signaling pathways such as the Wnt/Ca^2+^ route and the planar cell polarity (PCP) pathway (88). When Wnt5a is activated, a cascade of events begins, including PLC-mediated calcium signaling, CaMKII activation, and NFAT activation. Finally, it causes gene transcription and DNA replication in granulosa cells. Similarly, the PCP pathway regulates cytoskeletal changes and cellular polarity through RhoA and JNK, which helps organize tissue in growing follicles (89).

The noncanonical Wnt signaling pathways, particularly the Wnt5a-mediated calcium (Ca^2+^) signaling, are crucial for various cellular processes, including cell migration, polarity, and differentiation (76, 90). The canonical Wnt pathway primarily functions through the stabilization and translocation of *β*-catenin to the nucleus (91). In contrast, noncanonical Wnt signaling operates independently of β-catenin, activating alternative intracellular signaling pathways, such as the calcium signaling pathway and the planar cell polarity (PCP) pathway (92). Wnt5a, a crucial ligand in the noncanonical pathway, is associated with the regulation of follicular development, especially during the transition from primary to small white follicles in avian species (75, 93). Wnt5a signaling may affect cellular processes, including cortical granule exocytosis, intercellular communication, and the regulation of growth factors that influence follicle maturation (94). The interaction between noncanonical and canonical Wnt pathways is complex (95). Research indicates potential crosstalk, wherein noncanonical signals may enhance or inhibit *β*-catenin-dependent transcriptional activation of target genes (96, 97). Interactions are essential for maintaining equilibrium among cell proliferation, differentiation, and apoptosis throughout follicular development (98). Examining the convergence and divergence of these pathways in regulating follicular dynamics may yield significant insights into reproductive biology and the molecular mechanisms that govern oogenesis in chickens.

This signaling network closely coordinates with other pathways, such as MAPK, TGF-β, and p53, as indicated by the KEGG enrichment analysis pathway maps, which precisely regulate cell cycle progression and maintain cellular homeostasis (99). This interaction ensures that the follicle grows coordinately in response to both internal and external stimuli (99). During the early stages of follicle growth, the Wnt/β-catenin/TCF-LEF signaling axis functions as a molecular switch, facilitating granulosa cell proliferation and the establishment of a structural framework (73, 75). A potential target for altering bird ovarian function could be its regulated activation during the transition from PM to SW follicle stage, emphasizing its critical involvement in folliculogenesis.

### PLB1 glycerophospholipid metabolism

Cellular activity increased during the development of ovarian follicles in chickens, from the SW to the SY phases. This activity includes metabolic remodeling, hormone generation, and membrane biosynthesis (100). This study highlights the physiological relevance of the PLB1-mediated glycerophospholipid metabolism pathway at this stage of follicle development by indicating a substantial enrichment of this pathway (Figure 8D). Phosphatidylcholine (PC) and phosphatidylethanolamine (PE) are glycerophospholipids that are involved in membrane structure and are also essential in signaling, cell proliferation, and cell differentiation (101, 102). Using the intermediates monomethyl-phosphatidyl-ethanolamine and dimethyl-phosphatidyl-ethanolamine, phosphatidylcholine (PC) is produced from phosphatidyl-ethanolamine (PE) in this process, which is facilitated by PE N-methyltransferases (103). This conversion is crucial for increasing membrane mass (87) to stimulate follicular cell growth and the ovulation process, as well as for maintaining lipid balance (104) in rapidly proliferating granulosa cells.

The Kennedy pathway is an alternative and supplementary pathway that produces phosphatidylcholine by phosphorylating choline to phosphocholine and then converting it to CDP-choline (105). During the metabolically active SY follicular stage, this branch is critical for cell proliferation, hormone production, and lipid droplet formation (106). As granulosa cells prepare for further expansion and steroidogenic differentiation, an upregulation of CDP-choline and related enzymes suggests an increased demand for membrane lipid production (107). The biosynthesis of phosphatidylethanolamine (PE) from ethanolamine involves using two intermediates, phosphoethanolamine and CDP-ethanolamine, in addition to PC biosynthesis (108). Enzymes linked to the CDP-choline and phosphatidylcholine N-methyltransferase (PEMT) pathways are upregulated during yolk deposition, indicating an increase in metabolism (109). To start vitellogenesis in SY follicles, these enzymes are crucial for lipid droplet production and transport (110, 111). The engagement of these lipid pathways enables the systemic regulation of folliculogenesis, as they meet the demand for estrogen-mediated yolk protein synthesis (112).

This PE branch plays a crucial role in mitochondrial function, apoptosis regulation, and energy metabolism (106), all of which are essential for oocyte maturation and follicle selection (113). CDP-ethanolamine levels significantly impact follicular activity and biosynthetic efficiency (114). The metabolic interactions that facilitate energy production and anabolic development are explained by the relationship between this route and acetaldehyde synthesis, which is linked to glycolysis and pyruvate metabolism (115). Enhanced glycolytic activity may meet the increased ATP demands of granulosa cell development and yolk storage in developing follicles (116, 117). Follicles undergo lipid remodeling and energy integration through the PLB1 glycerophospholipid metabolism pathway during the transition from the SW to the SY development phase. This mechanism relies on the transition from follicle development, membrane biosynthesis, hormone synthesis, and metabolic activity. The relevance of this pathway in establishing early follicular development in chickens is shown by the dynamic regulation of this pathway in our transcriptome studies.

Follicle development is not regulated by a single signaling pathway but rather by a highly intricate and coordinated network involving various molecular mechanisms, such as growth factor signaling (e.g., AKT/mTOR), morphological factors (e.g., Wnt), and metabolic reprogramming (e.g., pyruvate carboxylase metabolism). These pathways interact continuously throughout the maturation process of the follicle, ensuring that both granulosa cells and oocytes receive the necessary molecular processes for proper development. Transcriptomic analyses indicated that key transitions between developmental stages are controlled by the upregulation of specific genes and the selective downregulation of inhibitory factors, including PTEN, GSK3*β*, and SFRP1/5. These changes reflect the removal of inhibitory checkpoints, thereby enabling the full activation of the corresponding signaling pathways. Such regulation ensures that follicular progression occurs when both external and internal signals are appropriately synchronized. The findings offer crucial insights into the mechanisms of chicken follicle growth and laying a foundation for future advancements in avian reproductive research. The identification of stage-specific pathways linked to follicle development, growth, and selection offers opportunities for developing novel molecular strategies to enhance reproductive efficiency in poultry.

## Conclusion

This research presents a detailed molecular characterization of the signaling pathways in developing chicken ovarian follicles. Transcriptomic analysis revealed essential molecular mechanisms and pathways that govern follicular activation, growth, and maturation. The PI3K-Akt/mTOR and Wnt/β-catenin signaling pathways play a crucial role in the initial phases of follicular development by facilitating cell proliferation and survival. As follicles mature, metabolic pathways associated with cellular differentiation, growth, steroidogenesis, lipid metabolism, and extracellular matrix remodeling become increasingly significant, enabling the successful transition from SW to SY follicles. These findings highlight the intricate interplay between signaling and metabolic pathways involved in ovarian follicle development in chickens, providing crucial insights into the molecular mechanisms that regulate reproductive efficiency in avian species.

## Data Availability

The datasets presented in this study can be found in online repositories. The names of the repository/repositories and accession number(s) can be found in the article/Supplementary material.
