# Supplementary material for: Molecular profiling of signaling pathways involved in chicken ovarian follicle development by transcriptome sequencing
Source: Front Vet Sci. 2026 Jan 9;12:1676247. doi: 10.3389/fvets.2025.1676247 (PMC12827103; doi:10.3389/fvets.2025.1676247)
Supplement: Supplementary file 9 [file Table_9.docx]

**List of Primers for RTqPCR**

| No. | Gene | ID | Primer | bp |
| --- | --- | --- | --- | --- |
| 1 | AKT1 | >[NM_001396388.1](https://www.ncbi.nlm.nih.gov/entrez/viewer.fcgi?db=nucleotide&id=2117900769) | CCACACGCTTTCTGAGCAGC  GTCTTGCGGTCGTTCCTTGT | 531 |
| 2 | MTOR | >[XM_417614.8](https://www.ncbi.nlm.nih.gov/entrez/viewer.fcgi?db=nucleotide&id=2201656940) | CGAATCTGTAACGCTCCGCC  TCCTGGCTCATTTCACGGAG | 749 |
| 3 | RPS6KB1 | >[XM_046930143.1](https://www.ncbi.nlm.nih.gov/entrez/viewer.fcgi?db=nucleotide&id=2201759836) | CCTCTCCCAGGCTTTAAGGAA  TGGAGATGGGTGAGCAAACG | 469 |
| 4 | PTEN | >[XM_040674795.2](https://www.ncbi.nlm.nih.gov/entrez/viewer.fcgi?db=nucleotide&id=2201808835) | GCAGCCATGATGGGAGCGTA  ATTGCAAGTTCCGCCACTGA | 743 |
| 5 | CTNNB1 | >[XM_046910392.1](https://www.ncbi.nlm.nih.gov/entrez/viewer.fcgi?db=nucleotide&id=2201761939) | TCCAGGAATGAGGGTGTTGC  ATGATACAGCATCTGGGCGG | 211 |
| 6 | WISP1 | >[XM_046910739.1](https://www.ncbi.nlm.nih.gov/entrez/viewer.fcgi?db=nucleotide&id=2201762622) | CTGCAGCATATGAGGGGGAG  CCATCTGGGCACTCAAACCT | 376 |
| 7 | MAPK8 | >[XM_040703166.1](https://www.ncbi.nlm.nih.gov/entrez/viewer.fcgi?db=nucleotide&id=2024418667) | TGGTGCCAAACCAGATAGGG  GGATGACCTCTGGTGCTCTG | 715 |
| 8 | PCYT1A | >[XM_046898487.1](https://www.ncbi.nlm.nih.gov/entrez/viewer.fcgi?db=nucleotide&id=2201696736) | GAGTGAACATGGCTGCACGA  TCCAAAGGGGTCCCTCTCAT | 388 |
| 9 | ETNK1 | >[XM_416426.8](https://www.ncbi.nlm.nih.gov/entrez/viewer.fcgi?db=nucleotide&id=2201579768) | TGTTCTGCACCAGGTGGTTT  GAACCCGAGACAAACGCTCT | 212 |
| 10 | RBL2 | >[NM_001397481.1](https://www.ncbi.nlm.nih.gov/entrez/viewer.fcgi?db=nucleotide&id=2148832372) | AGCACAGGTAGGCCCTTCTC  TGAGTGCCTCGGTACAATGC | 446 |
| 11 | CDKN1B | >[NM_204256.3](https://www.ncbi.nlm.nih.gov/entrez/viewer.fcgi?db=nucleotide&id=2099375716) | CTGGAAGGCAGGTACGAGTG  TCCTCCGCCTTAGGGAGTTT | 408 |
| 12 | FOXO3 | >[XM_001234495.7](https://www.ncbi.nlm.nih.gov/entrez/viewer.fcgi?db=nucleotide&id=2201670471) | CTGGCACACCCCAAAATGTG  CGCTGTGGACATCACCCAT | 149 |
